# Ontogenetic comparisons of standard metabolism in three species of crocodilians

**DOI:** 10.1371/journal.pone.0171082

**Published:** 2017-02-09

**Authors:** C. M. Gienger, Matthew L. Brien, Christopher R. Tracy, S. Charlie Manolis, Grahame J. W. Webb, Roger S. Seymour, Keith A. Christian

**Affiliations:** 1 Research Institute for the Environment and Livelihoods, Charles Darwin University, Darwin, Northern Territory, Australia; 2 Wildlife Management International and Crocodylus Park, Berrimah, Northern Territory, Australia; 3 School of Earth and Environmental Sciences, University of Adelaide, Adelaide, South Australia, Australia; University of Tasmania, AUSTRALIA

## Abstract

Due in part to their large size, aggressive temperament, and difficulty in handling, there are few physiological studies of adult crocodilians in the literature. As a result, studies comparing individuals across an ontogenetic series and comparisons among species are also lacking. We addressed this gap in knowledge by measuring standard metabolic rates (SMR) of three species of crocodilians (*Crocodylus porosus*, *C*. *johnsoni*, and *Alligator mississippiensis*), and included individuals that ranged from 0.22 to 114 kg. Allometric scaling of SMR with body mass was similar among the species, but *C*. *porosus* had significantly higher SMR than did *C*. *johnsoni* or *A*. *mississippiensis*. Differences in SMR among species are potentially related to behavioural differences in levels of aggression; *C*. *porosus* are the most aggressive of the crocodilians measured, and have rates of standard metabolism that are approximately 36% higher at the grand mean body size than those measured for *C*. *johnsoni* or *A*. *mississippiensis*, which are among the least aggressive crocodilians.

## Introduction

Crocodilians (clade Crocodylia: Gavialidae, Alligatoridae, and Crocodylidae) have been an important group in our understanding of animal nutrition, growth, and physiology. As a result of their remarkable fecundity, rapid growth rates, and relative ease of captive propagation, crocodilians have long been a model for studies in reptilian nutritional physiology [1, 2]. However, a large majority of the published data regarding crocodilian physiology and metabolism has been collected from juveniles weighing less than ~3 kg, a size that is typically reached by most crocodilians within the first few years of life [3, 4]. The result is a potential demographic bias in the literature towards the study of young individuals that could yield a relatively myopic view of ontogenetic changes in physiological function that may accompany the extreme growth and large body sizes achieved by crocodilians. For example, the largest of the extant crocodilians, the saltwater crocodile (*Crocodylus porosus*), changes in body mass by more than four orders of magnitude across a lifetime from a ~60 g hatchling [5] to just over 1,000 kg in the very largest of adults [6].

Because physiological data for larger crocodilians are relatively scarce and because there are considerable logistic and safety justifications for focusing research efforts on smaller crocodilians rather than large, ontogenetic comparisons among species are largely lacking from the literature. This study was undertaken to partially address this deficiency by comparing how standard metabolic rate (SMR) changes with ontogeny in three species of crocodilians, *C*. *porosus*, *C*. *johnsoni*, and *Alligator mississippiensis*. These species are all among the largest of the extant crocodilians, but differ appreciably in temperament; *A*. *mississippiensis* and *C*. *johnsoni* are considered among the least aggressive of the crocodilians, where *C*. *porosus* is thought to be the most aggressive [7, 8] There is a growing body of literature linking variation in rates of energy use (both intra and interspecifically) to differences in behavioral phenotype and temperament [9–12], and we explore how potential differences in energy use among crocodilians may be related to temperament and aggressive behavior.

## Materials and methods

Our methods for measuring standard metabolic rates are described in detail in two previous studies [13, 14], but we briefly summarize them here. Crocodiles and alligators in this study (*C*. *porosus*, *C*. *johnsoni*, and *A*. *mississippiensis*) were from captive populations maintained by Crocodylus Park, Darwin, Northern Territory, Australia. Animals were housed in outdoor enclosures and were divided into conspecific groups containing other similar-sized individuals. Each enclosure had a pond area with water deep enough for swimming and retreat, and a dry-land area for basking. Body mass ranged from 0.22 to 114 kg. Animals less than 1 kg were weighed to ± 1 g using a laboratory scale, those between 1 and 25 kg were weighed to ± 10 g using a postal scale, and those above 25 kg were weighed to ± 1 kg using a crane scale.

For individuals less than 25 kg body mass, we used metabolic chambers made from opaque PVC pipes (250–400 mm diameter; 30–200 L) matched to body size. Chambers were filled approximately half full with water [15] and temperature was maintained at 30°C by submersed 100 W aquarium heaters. For the larger individuals (> 25kg), we used a single chamber, 4 m long, 1.5 m wide, and 1 m tall. The chamber was constructed from 6 mm plate steel, sealed at one end, and access was through a removable air-tight door bolted to a flange at the opposite end. Four 250 mm circular access portals on the top and sealed ends of the chamber allowed for filling of the tank and for visual inspection of crocodiles. The chamber was filled about two-thirds full with water and temperature was maintained at 30°C by four 500 W submersed aquarium heaters. The steel metabolic chamber and associated gas sampling equipment were housed inside a 6 m insulated and temperature controlled (25°C) commercial steel shipping container.

For safety reasons, it was necessary to incapacitate the larger crocodiles (> 10 kg) temporarily while they were transferred to and from respirometry chambers. We subdued larger individuals with electro-stunning, and a 12V charge was applied to the dorsal surface of the neck for 3 to 10 s (Graham Electrical Stunners, Atherton, QLD, Australia). Stunning temporarily incapacitates the animal so that the jaws can be secured shut and the eyes covered before the animal has a chance to struggle. Individuals typically remain immobilized for 5 to 10 min after stunning and usually return to normal activities (e.g. swimming and basking) soon after being released. Electro-stunning is a common handling practice in the alligator and crocodile farming industries [16] and is less stressful to the animals than using the alternative of manual restraint with ropes or webbing [17]. All experimental protocols were approved by the Charles Darwin University Animal Ethics Committee (project reference number A09015).

Rates of oxygen consumption were measured using flow-through respirometry [18, 19]. Room air was pulled through a drying column (silica gel) and passed through a mass flow meter (Sierra Instruments) and respirometry chamber. Flow rates were adjusted from 0.2 to 10 L min^-1^ according to the size of the animal. A subsample of excurrent chamber air was drawn through a drying column (Drierite; Hammond Co.) and into an oxygen analyzer (Fox Box, Sable Systems International or Ametek S-3AI, AEI Technologies). A gas flow switching system was programmed to alternate sampling between the excurrent chamber air stream (sampled for 45 min each hour) and a baseline stream of dry room air (15 min each hour). Oxygen concentration was recorded every 3 s on a laptop computer using LabHelper software (Warthog Systems).

Individuals were fasted for 3–4 days before being moved into respirometry chambers, and after a settling-in period of 6–12 h, each was measured for 3–4 days. Rates of oxygen consumption were calculated using standard equations [20], implemented in LabAnalyst (Warthog Systems) that used the most level 15 min section (300 samples) of each 45 min interval. The lowest recorded value during the entire run was taken as the standard metabolic rate (SMR).

**Fig 1:**
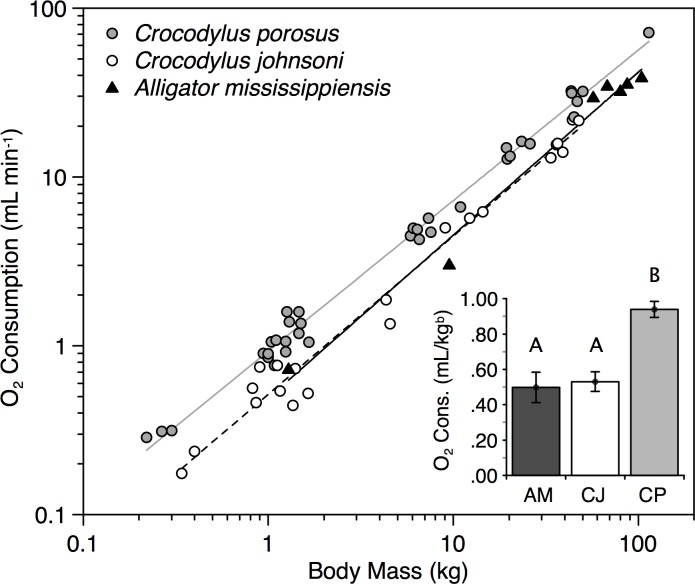
Scaling of standard metabolism (O_2_ consumption) for three species of crocodilians measured at 30°C. The highly aggressive *Crocodylus porosus* has significantly higher standard metabolism than the less aggressive *Crocodylus johnsoni* and *Alligator mississippiensis*. Inset figure shows mass-independent means (bars) and standard errors (error bars) of O_2_ consumption. Species not connected by same letter are significantly different.

We attempted to use animals of similar size ranges for each of the three species measured (Fig 1). However, this was complicated by two factors: that adult *C*. *porosus* grow much larger than *C*. *johnsoni* and *A*. *mississippiensis*, and that we did not have access to individual *A*. *mississippiensis* < 9.5 kg. In order to balance the ranges of body sizes used to compare species we limited measurements to individuals ≤ 114 kg, and supplemented the dataset with published values from the literature. We used data from Lewis and Gatten (1985) for four juvenile *A*. *mississippiensis*, and data in that study were collected in a similar fashion as our measurements (standard metabolism at 30°C using metabolic chambers partially filled with water). Data from Lewis and Gatten [21] were reported in mass-specific units, and we calculated mean whole-animal rate from the reported mean body mass (1.29 kg).

Rates of oxygen consumption (SMR) and body mass data were log_10_-transformed so that conventional allometric equations could be derived from ordinary least squares regressions [22]. The equations have the form SMR = *aM*^*b*^, where *a* is the scaling coefficient (intercept), *M* is body mass, and *b* is the exponent (slope of the regression line). Differences in scaling coefficients and exponents were tested with analysis of covariance (ANCOVA), according to Zar [23]. Log_10_ body mass was used as a continuous covariate in comparison of log_10_ SMR among species (categorical predictor variable).

Because the range and distributions of body sizes differ among species, we also compare mass-independent SMR among species using ANOVA. We divided whole-animal metabolic rate by the body mass of each individual raised to its species-specific scaling exponent (mass-independent SMR = whole-animal SMR/*M^*^*b*^). This removes the allometric effect of body mass on SMR allowing a mass-independent comparison [24]. Pairwise differences in whole-animal SMR and mass-independent SMR between species were made using Scheffé post-hoc contrasts.

## Results

Standard metabolic rate (ml O_2_ min^-1^) was measured in 63 individuals: 35 *C*. *porosus* ranging in size from 0.22 to 114 kg, 21 *C*. *johnsoni* ranging from 0.34 to 48 kg, and 7 *A*. *mississippiensis* ranging from 1.29 to 104 kg (Fig 1). Standard allometric equations relating body mass to SMR at 30°C are SMR = 0.931*M*^0.892^ for *C*. *porosus* (standard error of exponent = 0.015), 0.516*M*^0.937^ for *C*. *johnsoni* (s.e. = 0.032), and 0.491*M*^0.965^ for *A*. *mississippiensis* (s.e. = 0.054). The slopes of the allometric relationships between body mass and SMR did not differ among species (no significant interaction; F_2,59_ = 1.7, P = 0.19), but there was a significant difference in intercepts among species (ANCOVA F_2,59_ = 51.8, P < 0.001). Least square means of SMR were 4.26 (s.e. = 0.033) mL O_2_ min^-1^ for *C*. *porosus*, 2.53 (s.e. = 0.042) mL O_2_ min^-1^ for *C*. *johnsoni*, and 2.77 (s.e. = 0.077) mL O_2_ min^-1^ for *A*. *mississippiensis*. On a pairwise basis, *C*. *porosus* had a higher SMR than both *C*. *johnsoni* and *A*. *mississippiensis* (Scheffé S = 95.7 and 25.5 respectively, and P < 0.001 for both), but *C*. *johnsoni* and *A*. *mississippiensis* did not differ from one another (S = 1.1, P > 0.5).

Mass-independent means of SMR were 0.94 (s.e. = 0.021) mL O_2_/kg^b^ for *C*. *porosus*, 0.53 (s.e. = 0.027) mL O_2_/kg^b^ for *C*. *johnsoni*, and 0.50 (s.e. = 0.047) mL O_2_/kg^b^ for *A*. *mississippiensis*. On a pairwise basis, *C*. *porosus* had a higher mass-independent SMR than both *C*. *johnsoni* and *A*. *mississippiensis* (Scheffé S = 11.6 and 8.4 respectively, and P < 0.001 for both), but *C*. *johnsoni* and *A*. *mississippiensis* did not differ from one another (S = 0.5, P = 0.85).

## Discussion

There is much variability in rates of crocodilian metabolism, even under standard conditions [13, 21]. The majority of variation in SMR can be explained by body size, but behavioural and ecological differences among species likely also contribute [25–28]. Our results are consistent with general physiological and behavioural hypotheses that link variation in animal temperament to variation in energy use [9–12]. Our data support the notion that SMR is correlated with temperament in crocodilians, interspecifically, and we propose it as a general hypothesis that is in need of testing.

The species measured in this study are at opposite ends of the spectrum in relative levels of aggression and in tolerance of conspecifics. While *A*. *mississippiensis* and *C*. *johnsoni* are among the least aggressive of the crocodilians, *C*. *porosus* is arguably the most aggressive [7, 8]. More measurements are needed, and we suggest some key species that should be measured to address the hypothesis; *C*. *mindorensis* (Philipine Crocodile) and *C*. *rhombifer* (Cuban Crocodile) are also considered very aggressive (G.J.W. Webb personal observation) while *C*. *siamensis* (Siamese Crocodile) and *Gavialis gangeticus* (Gharial) are thought to be among the least aggressive [8].

The correspondence between aggressive-dominance behaviour and energy use has been demonstrated intraspecifically in other species, including Atlantic salmon [29, 30], brown trout [31], and giant freshwater prawns [32]. Lizards that had levels of aggression experimentally increased by testosterone implants showed rates of field metabolism that were elevated compared to control individuals (Marler and Moore 1989). However, increased energy expenditures were primarily the result of increased aggression and territory defence rather than elevated SMR alone [33]. Thus, the energetic costs of increased aggression may be manifested in both elevated SMR and through increased energy expenditure due to behavioural interactions.

The differences in SMR among *C*. *porosus*, *C*. *johnsoni*, and *A*. *mississippiensis* are considerable, and at a body mass of 20 kg (the approximate mean body size of all measured individuals) differences amount to a 36% higher standard metabolic requirement for *C*. *porosus* relative to the other species. However, comparison of SMR at the mean body size is somewhat confounded by ontogenetic effects, and at 20 kg the three species are at different life stages; *C*. *johnsoni*, and *A*. *mississippiensis* are near or at sexual maturity, whereas *C*. *porosus* is not sexually mature until reaching a much larger size [34, 35]. Therefore, higher SMR in *C*. *porosus* over the size range examined here might be partially explained by higher than expected metabolic rates that frequently accompany rapid juvenile growth in reptiles [36–38].

This report fills an important gap in the literature, and is the first report to detail comparative energetics of crocodilians across a significant portion of their natural size range. Because SMRs of the majority of crocodilian species (>23) have not been examined, the analyses presented here should provide a good foundation for additional studies that consider the energetic consequences of behavioural and ecological differences among the crocodilians.

## Supporting information

S1 File Raw data used to calculate standard metabolism in three species of crocodilians. (XLSX)
